# Preserved Nephrogenesis Following Partial Nephrectomy in Early Neonates

**DOI:** 10.1038/srep26792

**Published:** 2016-05-31

**Authors:** Yuhei Kirita, Daisuke Kami, Ryo Ishida, Takaomi Adachi, Keiichi Tamagaki, Satoaki Matoba, Tetsuro Kusaba, Satoshi Gojo

**Affiliations:** 1 Department of Nephrology, Graduate School of Medical Science, Kyoto Prefectural University of Medicine, 465 Kajii cho, Kamigyo ku, Kyoto 602-8566, Japan; 2 Department of Regenerative Medicine, Graduate School of Medical Science, Kyoto Prefectural University of Medicine, 465 Kajii cho, Kamigyo ku, Kyoto 602-8566, Japan; 3 Department of Cardiovascular Medicine, Graduate School of Medical Science, Kyoto Prefectural University of Medicine, 465 Kajii cho, Kamigyo ku, Kyoto 602-8566, Japan.

## Abstract

Reconstitution of total nephron segments after resection in the adult kidney has not been achieved; however, whether the neonatal kidney can maintain the capacity for neo-nephrogenesis after resection is unknown. We performed partial resection of the kidney in neonatal rats on postnatal days 1 (P1x kidney) and 4 (P4x kidney) and examined morphological changes and relevant factors. The P1x kidney bulged into the newly formed cortex from the wound edge, while nephrogenesis failure was prominent in the P4x kidney. Twenty-eight days post-resection, the glomerular number, cortex area, and collecting duct were preserved in the P1x kidney, whereas these parameters were markedly decreased in the P4x kidney. During normal development, *Six2* expression and Six2+ nephron progenitor cells in the cap mesenchyme both rapidly disappear after birth. However, time course analysis for the P1x kidney showed that *Six2* expression and Six2+ cells were well preserved in the tissue surrounding the resected area even 2 days after resection. In conclusion, our results indicate that kidneys in early neonate rats retain the capability for neo-nephrogenesis after resection; however, this ability is lost soon after birth, which may be attributed to a declining amount of Six2+ cells.

Regeneration is characterized as a process of renewal, restoration, and reformation of the tissue that is lost due to various insults, and it has been investigated for several hundreds of years in various species^1^. All species of animals and plants possess the capacity for regeneration, but abilities are diverse among species, tissues, cells, and aging stages. For example, after limb amputation in salamanders, exposed tissues are immediately covered by epithelium, beneath which undifferentiated cell aggregates known as blastema are generated. This is followed by complete limb regeneration through differentiation of cells in the blastema into various cell types^2^,^3^. This phenomenon is not observed in adult mammals, although murine neonatal fingertips can be regenerated following amputation^4^. In mammals, some tissues that undergo consistent cell loss (e.g. the intestine) have an adult stem cell population and continuously replace differentiated cells to maintain tissue homeostasis^5^. In contrast, some tissues such as the heart, lung, and kidney exhibit a much lower rate of cell turnover^6^. Lineage analysis of these tissues has shown that even after injury and repair, the contribution of the stem/progenitor population, if it exists, to organ regeneration is quite small^7^,^8^.

Our society is aging worldwide, and the number of patients with end-stage organ failure involving the heart, lung, and kidney, as well as the cost of treating these diseases, is increasing. These factors have a significant impact on individuals, public health, and the medical economy^9^. The histology of various tissues from end-stage organ failure is commonly characterized as the loss of normal cells and tissue structure, which are replaced by fibrous tissues that are responsible for the loss of organ function. The development of organ failure is generally attributed to an imbalance or impairment due to injury. After insults that cause persistent inflammation (e.g. resection, toxin exposure, and ischemia oxidative stress) subsequent regeneration processes activate pro-fibrotic signaling pathways and extracellular matrix deposition produced by activated fibroblasts, known as myofibroblasts^10^. Regenerative medicine encompasses interventions that are used to accelerate regenerative process *in situ* and the use of tissue engineering to treat disorders including organ failure; these promising therapeutic approaches have shown curative potential in several diseases^11^.

Treatments based on cell transplantation, however, have shown unsatisfactory results, and the field of regenerative medicine is still in its infancy. Regenerative medicine for the treatment of heart injuries is the most investigated modality among the various organs. Cell transplantation to damaged hearts, including resident progenitors^12^ and bone marrow-derived stem cells^13^, has gained attention, but modest improvements in pathophysiology and safety profile require additional mechanistic analyzes^14^,^15^,^16^, such as whether organogenesis can be recapitulated or inflammation can be ameliorated ubiquitously in failed organs, regardless of the etiology. In cell transplantation to treat kidney injury, exogenous stem cell injection of mesenchymal stem cells^17^, bone-marrow derived stem cells^18^,^19^ or renal progenitor candidates^20^,^21^ enabled these cells to engraft into the kidney and ameliorate the injury, but these cells rarely migrated and transdifferentiated into tubular epithelia. Lineage analysis of terminally differentiated tubular epithelial cells also excluded the contribution of intratubular progenitors and endogenous progenitors to the repair process in rodents^7^,^22^. These results indicate that the effect of cell therapy on kidney injury is not attributed to cell transdifferentiation into mature proximal tubular epithelia, but rather to paracrine systems^23^,^24^.

Developmental processes include cell proliferation, region specification, and differentiation into site-specific mature cells, which are similar to the regenerative process to some extent. Studies of developmental processes have investigated which of these processes are applicable to adult regeneration therapy. Although epimorphic regeneration has been limited to non-mammalian vertebrates, the neonatal period can shed light on regeneration processes, since both diminution of nephrogenesis and acquisition of mature renal structure and function are simultaneously proceeding. Murine neonates were exposed to cardiac apex resection at one week after birth, which resulted in pre-existing cardiomyocyte dedifferentiation and proliferation with little fibrosis^25^. Like newt limb regeneration, both dedifferentiation and progenitors/stem cells contributed to cardiac apex regeneration^26^. Murine neonatal kidneys exhibited Six2+ cells within the outer layer of the cortex at birth, the levels of which sharply decreased by 4 days of age^27^. In this study, we hypothesized that the neonatal kidney could maintain the capability to generate new nephrons until 4 days after birth. Neonates may offer insight into retrieving the capability of nephrogenesis during adulthood. Moreover, pediatric and adolescent renal disorders may also be reverted similarly to neonatal regeneration.

## Results

### P1x kidneys formed new cortex from the edge of the wound

**Figure 1:**
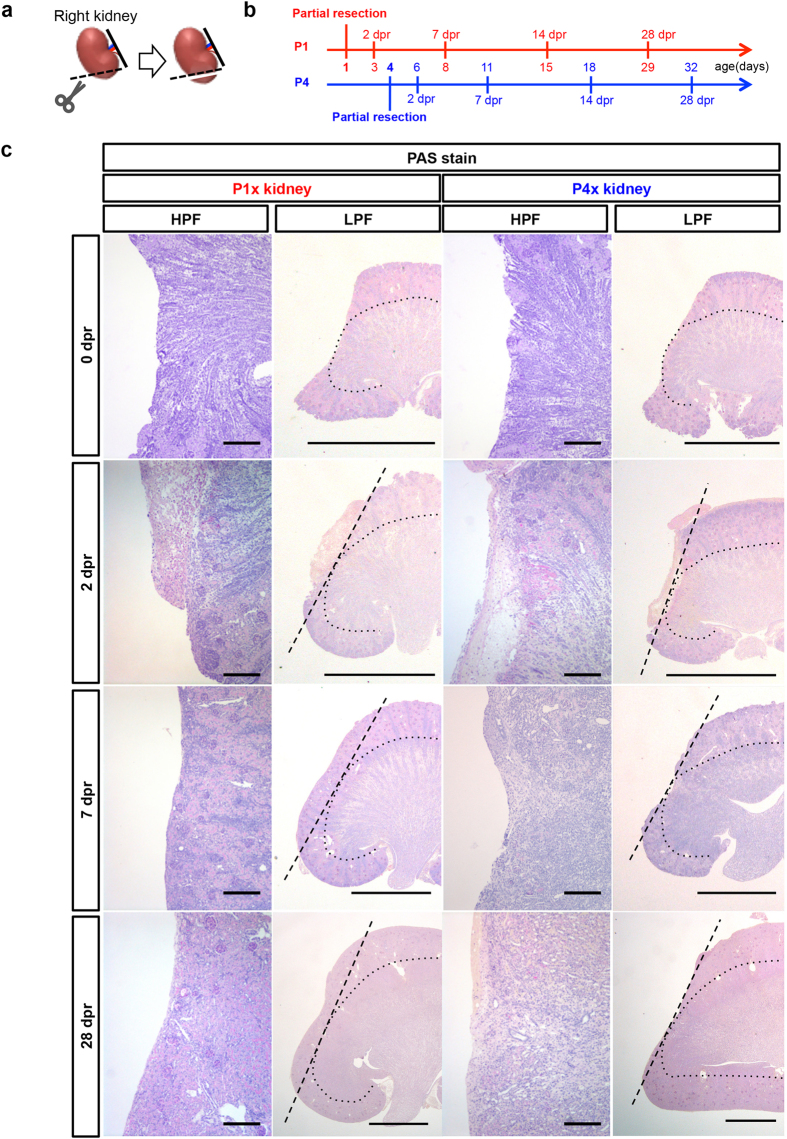
The P1x kidney generates the tissue from the edge of the wound after partial nephrectomy. (**a**) The right kidney at the inferior pole was partially resected in neonatal rats at postnatal day 1 (P1) or 4 (P4) under hypothermic anesthesia. An incision was made in the right flank and the right kidney was partially resected using iridectomy scissors. (**b**) Experimental scheme. After partial resection of the kidney, pups were sacrificed at 2, 7, 14, and 28 days post-resection (dpr). (**c**) Periodic Acid Schiff’s (PAS) staining of the kidney at 0, 2, 7, and 28 dpr. Dotted straight lines indicate the resection plane and dotted curves indicate the cortico-medullary junction (scale bars, 100 μm in HPF and 2 mm in LPF).

To examine the nephrogenesis capacity of the neonatal rat kidney after injury, we surgically resected the inferior pole of the right kidney (approximately 10% of the right kidney) from rats on postnatal day 1 (P1) and day 4 (P4) under hypothermic anesthesia (referred to as P1x and P4x kidney, respectively, Fig. 1a, Supplementary Movie S1). We examined kidney tissues from the rats at 2, 7, 14, and 28 days post-resection (dpr) (Fig. 1b).

The neonatal kidney has a complicated structure, and thus the depth of resection may affect the results. We validated the surgical procedure to ensure similar insult in both groups based on Periodic Acid-Schiff (PAS) staining of resected kidneys, which revealed that the resection reached the cortico-medullary junction in both P1x and P4x kidneys, and that the medullary tissue was exposed on the surface to some extent (Fig. 1c, Supplementary Fig. S1, 0 dpr). At 2 dpr in both P1x and P4x kidneys, macroscopic findings revealed that the surface of the resected site was covered with a blood clot, and PAS staining showed similar mononuclear cell infiltrations (Fig. 1c, Supplementary Fig. S1, 2 dpr). Subsequently, the parenchymal tissues around the edge of the wound gradually started to grow towards the center of the resected surface, and the bulk of the resected tissue was re-covered with vestiges, such as a flattened (not convex) surface by parenchymal tissue in the P1x kidney at 28 dpr (Fig. 1c, Supplementary Fig. S1, 28 dpr). However, in the P4x kidney, the remaining parenchymal tissues did not bulge over the resected site (Fig. 1c, Supplementary Fig. S1, 28 dpr). PAS, hematoxylin and eosin staining of the kidney sections from each time point showed that the resected site was gradually covered by tissues containing a number of tubules, and that mononuclear cell infiltration appeared to subside over time in the P1x kidney. In contrast, the resected site was not completely covered, resulting in scar formation with significant cell infiltration at 28 dpr in the P4x kidney (Fig. 1c, Supplementary Fig. S2). Immunostaining showed that large amounts of CD68 positive macrophages were found at 2 dpr in both P1x and P4x kidneys, but the infiltration of these cells was prolonged until the later time points in the P4x kidney (Supplementary Fig. S3).

### Nephrogenesis occurred in P1x kidneys, but not in P4x kidneys

**Figure 2:**
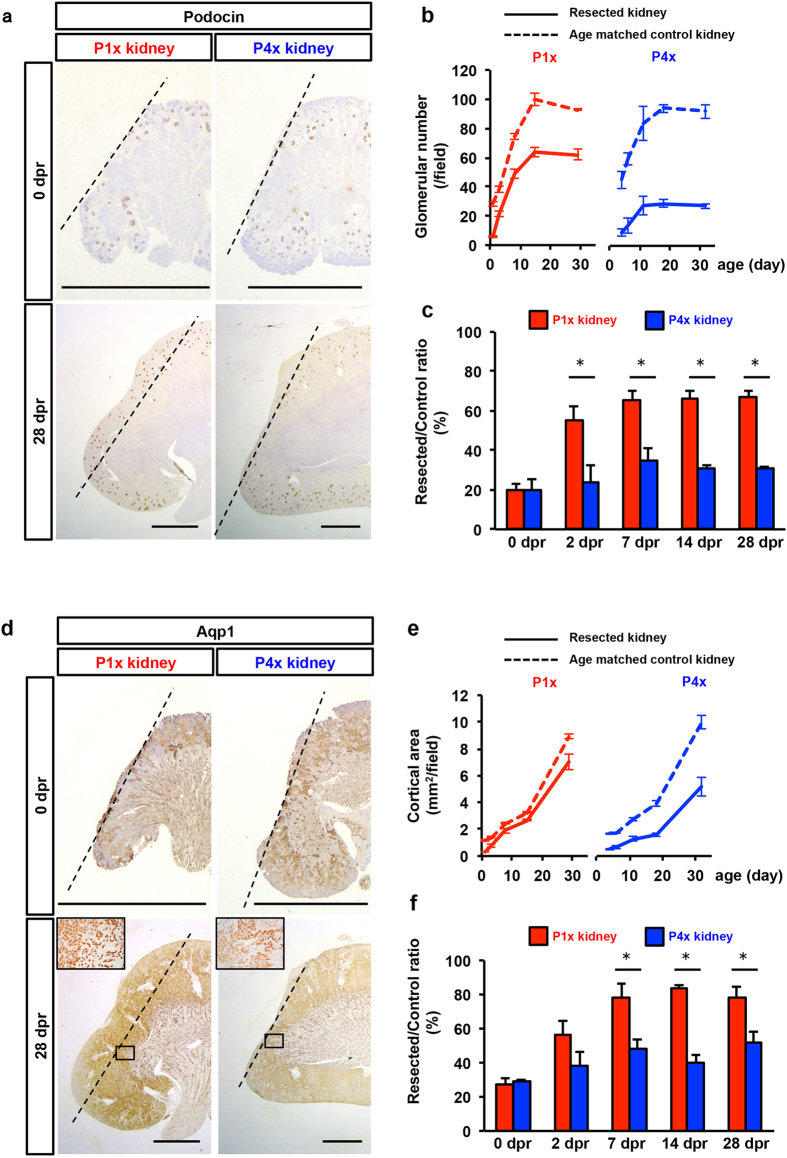
Newly formed glomeruli and tubules after partial nephrectomy of the P1x rat kidney. (**a**) Immunostaining of podocin as a glomerular marker. Dotted lines indicate the resection plane (scale bars, 2 mm). In the P1x kidney, the tissue covering the resected area contained glomeruli. In contrast, the newly generated tissue of the P4x kidney did not contain the glomerulus. (**b**) Time course analysis of glomerular number under the resection plane in the maximal cross-sectional surface. (**c**) Resected/control ratio of glomerulus number. (**d**) Immunostaining of AQP1 as a proximal tubule marker. Dotted lines indicate the resection plane (scale bars, 2 mm) and high magnification pictures are shown in small squares. In the P1x kidney, the tissue covering the resected area contained robust AQP1+ tubules, whereas this was not seen in the P4x kidney (**e**) Time course analysis of the cortical area under the resection plane in the maximal cross-sectional surface. (**f**) Resected/control ratio of cortical area. Data represent mean ± SE (n = 3). *P < *0.05.*

The nephron consists of the glomerulus, proximal and distal tubules, and collecting duct. We next examined which segments of the nephron contained generated cortical tissues. The generated tissues contained many mature glomeruli that were positive for podocin, a marker of mature podocytes, in the P1x kidney (Fig. 2a, Supplementary Fig. S4). PAS staining and immunohistochemical podocin expression showed structure similarity between the generated and developing glomeruli at the resected site and that of pre-existing glomeruli at a normal site (Supplementary Fig. S5). In addition, the tubules in the generated tissue were positive for aquaporin 1 (AQP1), a marker of mature renal proximal tubular epithelia (Fig. 2d, Supplementary Fig. S4). In contrast, these mature structures were less observed at 28 dpr in the P4x kidney (Fig. 2d, Supplementary Fig. S4).

We quantified the amount of mature proximal tubular epithelia and the glomerular number in the generated tissue of the kidney. At the later time points, as the edge of the resected site became unclear due to the growth of generated tissue, we determined the area to quantify glomeruli and tubules over the wound surface as follows. Immediately after nephrectomy in both P1x and P4x kidneys, the angle subtended by the lateral and medial edges of the wound line, with the center of the renal pelvis as the vertex, in the coronal slice with maximal cross-sectional area, was approximately 80 degrees, and this was not significantly different between the two groups. (Supplementary Fig. S6). Following the experiments, based on the line of the center point of the renal pelvis (as the endpoint) and the mid-point of the resected surface line, two rays at a 40-degree angle on both sides at the endpoint formed a fan-shaped area with the wound surface line. The glomerular number in the P1x kidney increased until 14 dpr at a similar rate as the age-matched control, reaching 66.7% of the control by 28 dpr (Fig. 2b,c). In contrast, the P4x kidney showed a slow rate of increase and an early plateau after 7 dpr, reaching only 35.0% of the control (Fig. 2b,c). The ratios of the glomerular number in the experimental kidneys to those in the control kidneys were significantly different at each observation point between the P1x and P4x kidneys. Moreover, the P1x kidney exceeded the glomerulogenesis of the control kidney until 7 dpr, and the increased rate in the early phase (0–2 dpr) was greater than that in the later phase (2–7 dpr). However, the P4x kidney did not significantly outstrip the control kidney (Fig. 2c), suggesting that amputation-induced neo-glomerulogenesis accelerated postnatal glomerulogenesis in normal development based on their similar kinetics.

In order to quantify tubulogenesis, cortical areas were examined using Aqp1+ cells as the marker (Fig. 2d). The cortical areas showed more robust nephrogenesis capabilities in the P1x kidney than in the P4x kidney compared with neo-glomerulogenesis. All groups showed increasing lines with similar slopes based on post-natal days (Fig. 2e), but the ratio of the cortical area in the experimental group to that of the control reached 77.9% versus 52.1% at 28 dpr, respectively (Fig. 2f). Neo-tubulogenesis exceeded the control kidney and showed the same kinetics as neo-glomerulogenesis, with a rapid early increase followed by later tapering of this effect.

**Figure 3:**
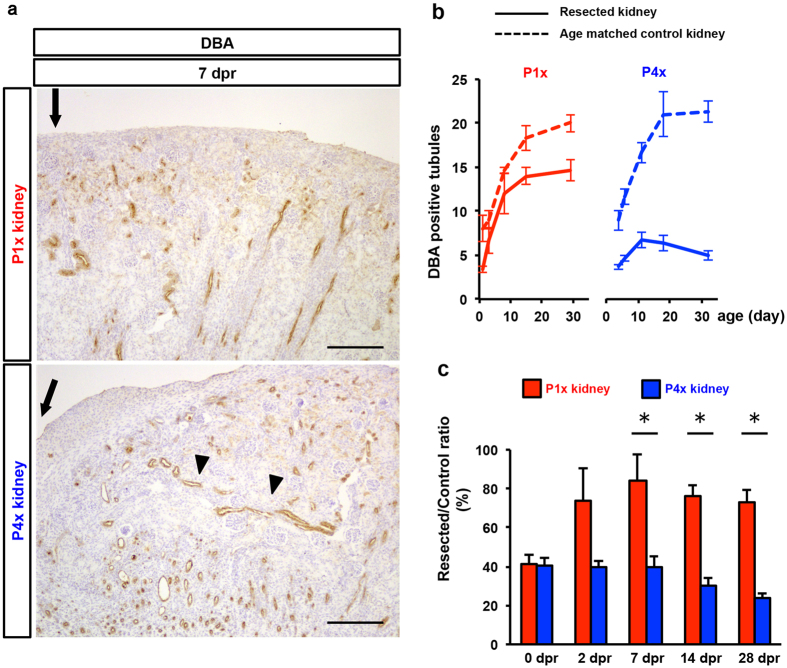
Newly formed collecting ducts after partial nephrectomy of the P1x rat kidney. (**a**) Immunostaining of DBA as a collecting duct marker. Arrows indicate the resected surface. Arrowheads indicate collecting ducts pulled into the resected area due to the scar constriction process in the P4x kidney. Scale bars, 100 μm. (**b**) Time course analysis of the number of DBA+ tubules crossing a boundary line between the cortex and medulla under the resection plane in the maximal cross-sectional surface. (**c**) Resected/control ratio of DBA+ tubules. Data represent mean ± SE (n = 3). *P < *0.05.*

We also evaluated the morphogenesis of the collecting duct, which were derived from the HoxB7 lineage, whereas the remaining nephron segments were derived from the Six2 lineage. Staining of *Dolichos biflorus* agglutinin (DBA), a general marker of developing renal collecting ducts, showed that perpendicular collecting ducts against the renal surface from the cortex to the papilla were prominent underneath the wound at 7 dpr in the P1x kidney (Fig. 3a). In contrast, DBA+ tubules at 7 dpr in the P4x kidney were sparse, and some of them did not align perpendicularly to the kidney surface (Fig. 3a). This difference suggests that the peripheral portion was connected to the lost cortex by the resection, and that the remaining collecting duct survived and consequently reconnected to newly generated tubules and glomeruli in the P1x kidney. In the P4x kidney, the remaining collecting duct could not subsist, and the collecting ducts in the cortex around the wound were pulled to the middle portion of our defined area because of the scar constriction process. We quantified the DBA+ tubules at the cortico-medullary junction in P1x and P4x kidneys (Supplementary Fig. S7). The number of DBA+ tubules showed similar kinetics to the glomerular number in the P1x kidney and the decreasing tendency after post-natal day 11 in the P4x kidney (Fig. 3b). For the time line of post-resection days, the ratio of the number of collecting ducts in the experimental kidney to the number in the control kidney made diminution in the P4x kidney clearer, and the new branching and elongation of remaining collecting ducts mainly evolved during the early phase, such as neo-glomerulogenesis (Fig. 3c). Because previous reports showed that retinoic acid signaling plays an indispensable role in renal organogenesis^28^,^29^, we examined the effect of exogenous retinoic acid administration on neo-nephrogenesis after resection in the neonatal kidney. Daily retinoic acid administration did not enhance neogenesis of the renal cortex, including in the glomerulus (Supplementary Fig. S8).

Taken together, these results indicate that the P1x kidney may still possess a robust capacity for neo-nephrogenesis of the renal cortex even after partial kidney resection, but this function is rapidly lost by 4 days after birth. All kinetics of neo-glomerulogenesis, neo-tubulogenesis, and new branching of the collecting ducts following the insult were very similar to normal development in the post-natal term, indicating that it is possible to manipulate post-natal nephrogenesis.

### Early neonatal rat kidneys showed proliferative and anti-apoptotic capacities

**Figure 4:**
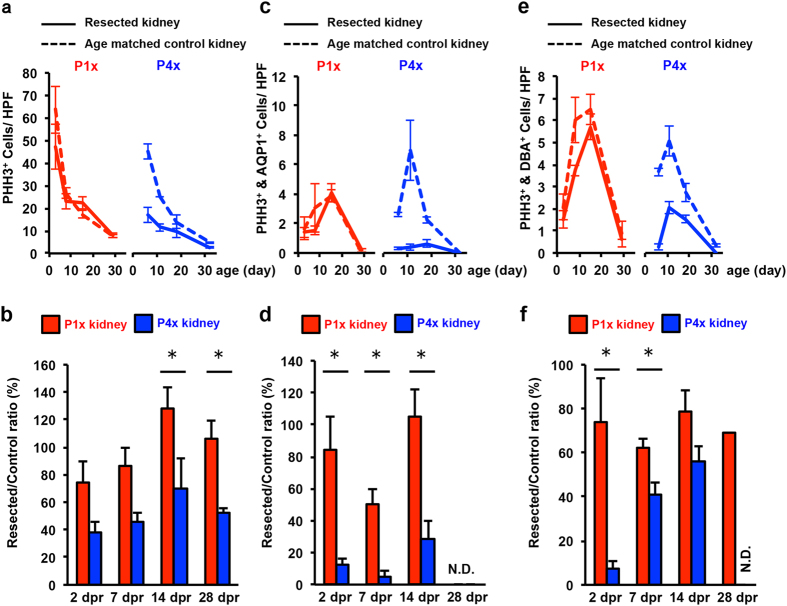
The P1x kidney preserves proliferative capacity after partial nephrectomy. (**a**) Time course analysis of the number of PHH3+ cells under the resection plane on the maximal cross-sectional surface. (**b**) Resected/control ratio of PHH3+ cells. (**c**,**e**) Time course analysis of the number of PHH3+ AQP1+ cells and PHH3+ DBA+ for P1x and P4x kidney. (**d,f**) Resected/control ratio of PHH3+ AQP1+ cells and PHH3+ DBA+. Data represent mean of sample averages ± SE (n = 3). *P < 0.05.

We examined whether proliferative capacity was preserved in the resected kidney using Phosphohistone H3 (PHH3) staining, which can detect cells undergoing mitosis. PHH3+ cells were scattered in both the cortex and medulla in both P1x and P4x kidneys (Supplementary Fig. S9). In P1x kidneys, the number of PHH3+ cells was similar to that in age matched control kidneys, except at 2 dpr (Fig. 4a,b). This suggests that a loss of the nephrogenic zone by partial nephrectomy decreased mitosis around the resection plane, but that nephrogensis restored proliferative capacity. In contrast, fewer PHH3+ cells in the P4x kidneys were observed compared to age-matched control kidneys (Fig. 4a,b). To further examine which nephron segments were responsible for proliferation, we performed co-staining of PHH3 and AQP1 or DBA. Co-staining of AQP1 and PHH3 showed that the amount of PHH3+/AQP1+ cells was similar between resected kidney and age-matched control for the P1x kidney. However, these cells were fewer in the P4x kidney (Fig. 4c,d and Supplementary Fig. S10a). Co-staining of DBA and PHH3 exhibited a similar phenomenon to AQP1 and PHH3 staining (Fig. 4e,f and Supplementary Fig. S10b). These results indicate that the nephrogenesis capacity of the neonatal rat kidney is lost within four days of postnatal life.

**Figure 5:**
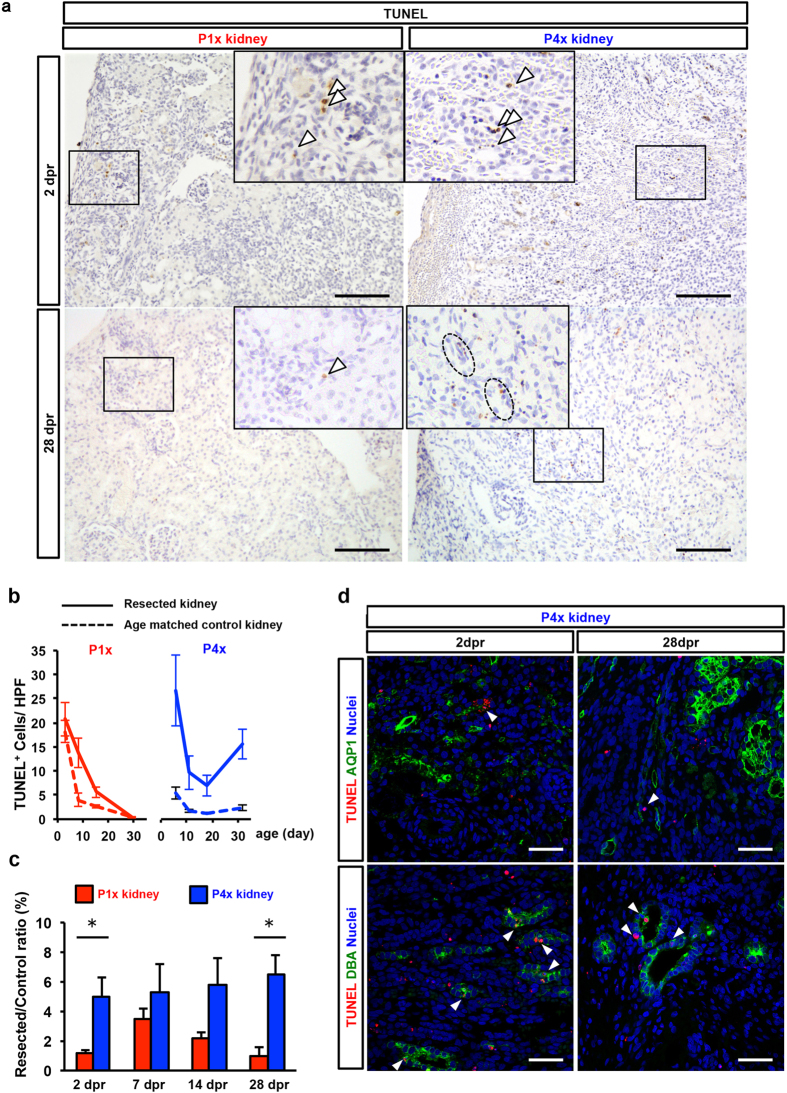
The P1x kidney preserves anti-apoptotic capacity after partial nephrectomy. (**a**) Immunohistochemistry of TUNEL for detection of apoptosis. Arrowheads indicate TUNEL+ cells. (**b**) Time course analysis of the number of TUNEL+ cells under the resection plane on the maximal cross-sectional surface. (**d**) Resected/control ratio of TUNEL+ cells. Data represent mean of sample averages ± SE (n = 3). *P < *0.05.* (**d**) Analysis of origin of apoptotic cells in the P4x kidney. Co-staining of TUNEL and AQP1 or DBA for detection of apoptotic cells at 2 dpr and 28 dpr in P4x kidneys. Scale bars, 100 μm.

We examined whether partial nephrectomy stimulates apoptosis around the resection plane. Apoptosis was assessed by counting terminal deoxynucleotidyl transferase nick-end labeling (TUNEL) + cells (Fig. 5a). In P1x kidneys, the number of apoptotic cells decreased over time, although the number was still greater than in control kidneys (Fig. 5b). The ratio of the number of apoptotic cells in P1x kidneys peaked at 7 dpr and converged to one at 28 dpr (Fig. 5c). In contrast, the number of apoptotic cells in P4x kidneys significantly increased over time (Fig. 5b,c) and were observed along nephrons (Fig. 5a); thus, partial nephrectomy at P4 caused nephron loss through growth. To analyze the origin of apoptotic cells in P4x kidneys, we performed the co-staining of TUNEL and AQP1 or DBA. AQP1+ TUNEL+ cells were rarely seen in P4x kidneys, but some TUNEL+ cells were positive for DBA (Fig. 5d). These results suggest that P1 rat kidneys have an anti-apoptotic capacity.

### Gene expression during normal kidney development on P1 and P4

**Figure 6:**
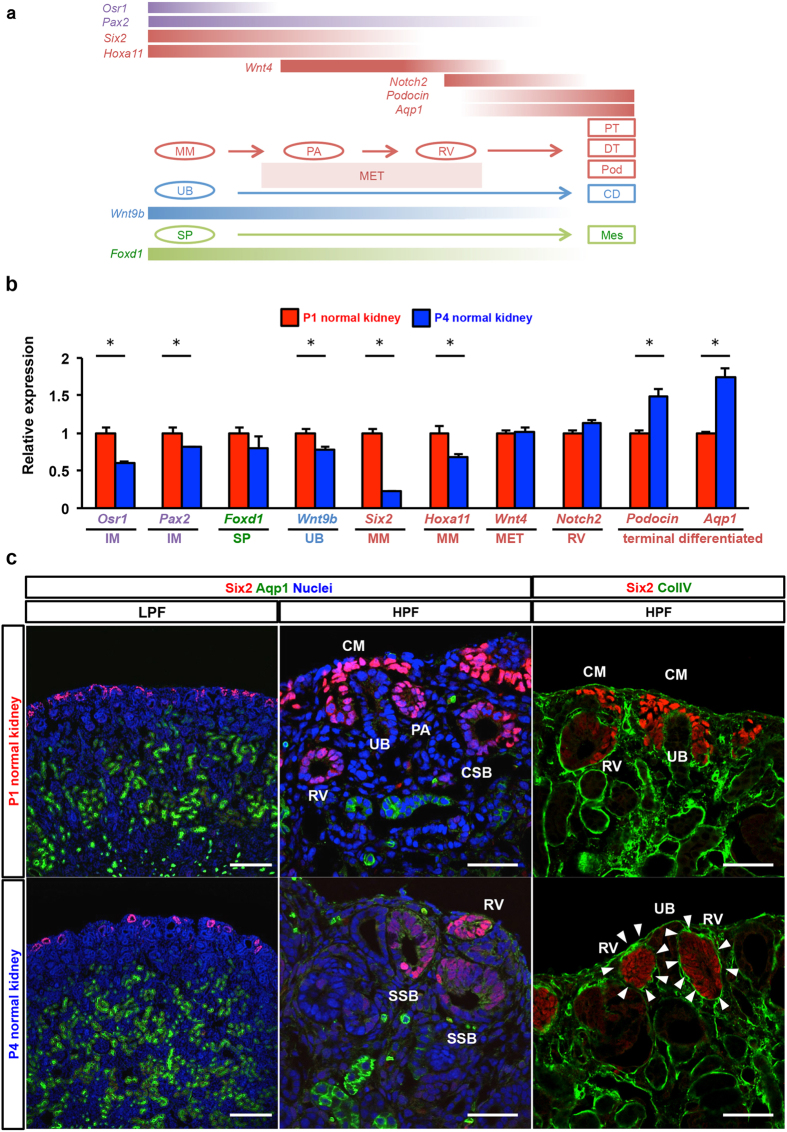
*Six2* gene expression and higher incidence of Six2+ cells in the normal kidney on P1 versus P4. (**a**) An illustration of temporal gene expression patterns during renal development. PS, pluripotent stem cell; IM, intermediate mesoderm; UB, ureteric bud; SP, Stromal progenitor cell; MM, metanephric mesenchyme; MET. Mesenchymal-epithelial transition; NP, nephron progenitor cell; RV, renal vesicle; DT, distal tubule; PT, proximal tubule; Pod, podocyte; CD collecting duct; Mes, mesangial cell. (**b**) The expression of marker genes for the developing kidney in P1 and P4 determined by qRT-PCR. The expression of each transcript relative to GAPDH expression is presented as the mean ± SE (n = 3) *P < 0.05. (**c**) Immunofluorescence staining for Six2 and Aqp1 in P1 and P4 kidneys. Scale bars in low power field (LPF), 200 μm; in high power field (HPF), 50 μm. Six2+ cells are present in the peripheral layer in both P1 and P4 normal kidneys; however, gene expression in the P4 normal kidney is much weaker than in the P1 normal kidney. Cap mesenchyme formation is observed in the P1 normal kidney but not in the P4 normal kidney. Immunostaining for collagen IV showed that renal vesicles are surrounded by a tubular basement membrane (white arrowheads), whereas this was not seen in the cap mesenchyme. CM, cap mesenchyme; PA, pretubular aggregate; RV, renal vesicle; CSB, Comma-shaped body; SSB, S-shaped body; UB, ureteric bud.

To further investigate the mechanisms relevant to the observed difference in the nephrogenesis capacity of resected kidneys, we performed gene expression profiling of P1 and P4 normal kidneys. We assessed various intermediate mesoderm-derived cell markers using qRT-PCR (Fig. 6a,b, and Supplementary Table S1). *Osr1* expression for the metanephric mesenchyme (MM) and *Pax2* expression for MM, pretubular aggregate (PA), renal vesicle (RV), and ureteric bad (UB) slightly decreased in the P4 normal kidney. *Foxd1* expression in the interstitial stroma and *Wnt9b* expression in the uretic bud were similar in both the P1 and P4 normal kidneys. *Six2* and *Hoxa11* expression in the MM was significantly lower in the P4 normal kidney compared to the P1 normal kidney (*P* < 0.05). To further analyze cap mesenchyme derivative markers, expression of *Wnt4* in the mesenchymal-epithelial transition, and *Notch2* in the renal vesicle (RV) and S-shaped body were not different between P1 and P4 normal kidneys. For terminally differentiated cell markers, *podocin* expression in mature podocytes and *AQP1* expression in the proximal tubule in the P4 normal kidney was 1.5-fold and 1.8-fold higher, respectively, than in the P1 normal kidney.

A drastic morphological change was observed within one week after birth, demonstrating that the cells derived from the Six2+ cap mesenchyme initially formed the RV, which is a primitive tubule containing the lumen and basement membrane^30^ during the mesenchymal-epithelial transition. Proximal-distal polarization is introduced within the RV thereafter, triggering fusion to the uretic stalk. We then evaluated the precise localization of Six2+ cells in the P1 and P4 normal kidneys. Immunofluorescence analysis showed that Six2+ cells were present at the peripheral layer in both P1 and P4 kidneys, but its expression level in P4 was much weaker than in P1 (Fig. 6c). Although *Six2*-expressing cells were observed in P4, the localization of these cells was restricted within the RV or S-shaped body, which is surrounded by the nascent tubular basement membrane (Fig. 6c). Cap mesenchyme formation, where the self-renewal of Six2+ nephron progenitors is maintained^31^, was observed in P1 but not in P4 (Fig. 6b). We co-stained the cells for AQP1, which is mainly expressed at the apical brush border of terminally differentiated proximal tubules and functions as a molecular water channel for reabsorption. In both P1 and P4 kidneys, robust tubular epithelia showed AQP1+ staining in the apical membrane, which was much more prominent in P4 kidneys (Fig. 6c). Since we suspected that the tubular base membrane directed Six2+ cell migration towards the wound, we visualized the tubular basement membrane by immunostaining of type IV collagen (ColIV). Co-staining with Six2 showed that the Six2+ cap mesenchyme in the P1 kidney never surrounded the tubular basement membrane. However, all RVs in both the P1 and P4 kidneys containing Six2+ cells were covered with basement membrane (Fig. 6c). To confirm the qPCR results for other markers, we also performed immunostaining of Pax2. The number of Pax2+ cells was much higher in P1 than in P4 kidneys, which was congruent with qPCR results (Supplementary Fig. S11).

### Partial nephrectomy in early neonates reinforced Six2 expression at the edge of the wound and generated new nephrons

We examined gene expression profiles of the markers for kidney development, inflammation, and tissue fibrosis in P1x and P4x kidneys over time following the resection by qPCR (Supplementary Fig. S12). Of note, the relative mRNA expressions of *Six2* and *Gdnf*, the ligand released from Six2+ cap mesenchyme during kidney development^32^, were greater at 2 dpr for P1x kidneys than those for the P4x kidneys. Based on the gene expression profiles and staining patterns of Six2 in P1 and P4 kidneys, we supposed that the Six2+ population might contribute to neo-nephrogenesis in P1x kidneys.

**Figure 7:**
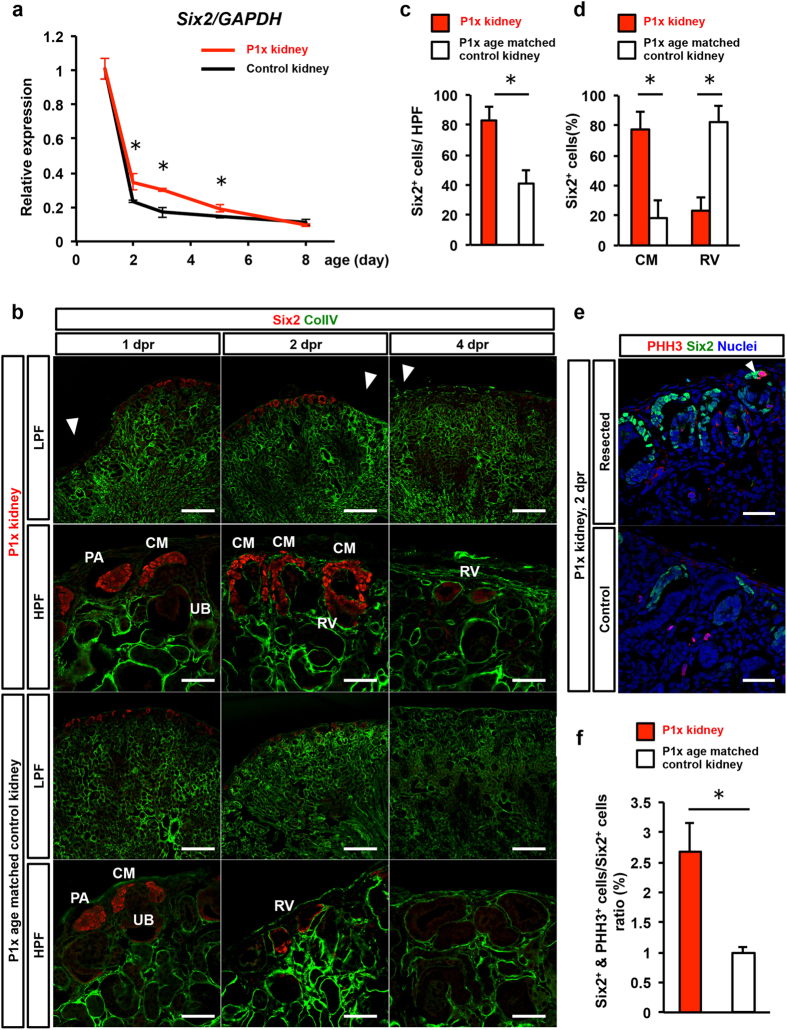
Partial nephrectomy in early neonates reinforces Six2 expression, preserves the cap mesenchyme structure in the edge of the wound, and generates new nephrons until 4 dpr. (**a**) Six2 expression dynamics in P1x and age-matched control kidneys determined by qRT-PCR. The relative expression of each transcript to GAPDH expression is presented as the mean ± SE (n = 3). Six2 expression rapidly decreases in the normal kidney; in contrast, the resected kidney preserves Six2 expression until 4 dpr. (**b**) Immunofluorescence staining of Six2 and ColIV in P1x and age-matched control kidneys. Arrowheads indicate the center of the resection plane. Six2+ cells localize on the anterior of newly generated tissues at 2 dpr, whereas they are scarcely observed in the age-matched control kidney. Six2+ cells disappear at 4 dpr in both resected and control kidneys. CM, cap mesenchyme; PA, pretubular aggregate; RV, renal vesicle. (**c**) The amount of Six2+ cells was the most prominent at 2 dpr in P1x kidneys. (**d**) The Six2+ cap mesenchyme was significantly preserved at 2 dpr in P1x kidneys. (**e**) Representative images of double staining for PHH3 and Six2 at 2 dpr. (**f**) Quantification of PHH3+ Six2+ cells showed that the number of cells was significantly larger in the P1x kidney.

We verified that the bulk of the multipotent Six2+ nephron progenitor population was lost around post-natal day 4, suggesting that the nephron number is eventually determined by 5 days after birth as previously reported^33^. Since previous lineage analysis showed that Six2+ cap mesenchymal cells are multipotent and can differentiate into podocytes and proximal and distal tubules^27^, we examined how the Six2+ population changed after resection and contributed to neo-nephrogenesis in the P1x kidney. Time course analysis by qRT-PCR showed that *Six2* expression was preserved more in the P1x kidney than in the normal kidney by post-natal day 5 (Fig. 7a). The well-preserved expression of *Six2* transcripts may reflect the larger number of cells expressing *Six2* rather than the increased intensity in pre-expressing cells, as demonstrated by the similar intensity observed in immunohistochemistry (Fig. 7b). Concerning the distribution of Six2+ cells, immunofluorescence analysis showed that Six2+ cells were localized on the front of generative tissues at 2 dpr (Fig. 7b, Supplementary Fig. S13), whereas only a few Six2+ cells were observed in age-matched control kidneys (Fig. 7b). We further quantified the number and the precise localization of Six2+ cells. The number of Six2+ cells was significantly higher in P1x kidneys. In addition, the bulk of Six2+ cells remained at the cap mesenchyme in P1x kidneys, while these cells localized to the RV in age-matched control kidneys (Fig. 7c,d). Finally, we compared the proliferative status of the Six2+ cells by co-staining PHH3 between P1x kidneys and age matched controls. The number of Six2+ PHH3+ cells was greater in the region surrounding the wound at 2 dpr for P1x kidneys compared to age matched controls (Fig. 7e,f). These results indicate that *Six2* expression was preserved, particularly on the front of the marginal zone of resection, and could act to maintain active proliferation and subsequent neo-nephrogenesis.

## Discussion

Renal development during the postnatal period has not been intensively investigated, particularly in regards to responses against various insults. We found that the capability to generate lost kidney tissue following insult by resection was preserved until postnatal day 4. The P1 neonatal kidney could rebuild the partially resected kidney with incomplete morphology, similar to a normally developed kidney. The existence of the Six2+ cell population in the cap mesenchyme may be a critical determinant, rather than other components, such as endothelial progenitors, to form the glomerulus and urinary buds that constitute the collecting duct systems. This neonatal partial nephrectomy model may shed light on not only the maturation process during the juvenile period, but also on the mechanism to generate a given number of nephrons in relation to renal disorder susceptibility.

Recently, nephrogenesis in mice has been clearly visualized using optical projection tomography, which revealed an array of 3-dimensional information to quantify volume, length, and angle in urinary branches, niches, cap mesenchyme cells, and total nephrons^34^,^35^. At the time of the parturition when urinary branching ceases in mice, approximately half of all nephrons present in adulthood are generated. The remaining nephrons are formed after birth between postnatal day 2 and 4^35^. The cap mesenchyme, in which the nephrons originate, enables the nephron progenitors expressing *Six2* and *Cited1* to form renal anlage^36^, which undergoes sequential transition from the pre-tubular aggregate, RV, comma-shaped body, to an S-shaped body as a consequence of the mesenchyme-to-epithelium transition^37^. Once the whole cap mesenchyme disappears by P4^33^, *de novo* nephrogenesis ceases, and the capacity to repair nephrons through dedifferentiation processes of existing constituents in the kidney is limited^7^,^38^,^39^,^40^. Unlike embryonic nephron formation, multiple (4–6) nephrons are formed at the single urinary tip during postnatal nephrogenesis^35^, and the sequence occurs at the periphery with existing nephrons sinking to the proximal portion^33^. How the bursting of nephrogenesis in this period is regulated and terminated remains unknown^37^. Nephrogenesis defines the number of nephrons, which varies from 100,000 to 1,000,000 in a single kidney in a healthy adult human. A reduction in the number of nephrons is a significant risk factor for hypertension and renal failure later in life^41^. There is no model available for examining the capacity of the cap mesenchyme to respond to stress during this period before their exhaustion. Neonatal partial nephrectomy, where the external insult to remove a part of the nephrogenic zone can induce supplemental nephrogenesis, should be an adequate experimental model for analyzing the cellular and molecular mechanisms occurring during the second phase of nephrogenesis. The supplemental nephrogenesis arose from the margin of the cutting edge on the nephrectomized kidney, and the pre-existing cap mesenchyme at the vicinity of the injury repopulated the defects to generate new nephrons through the persistent developmental process.

Our data demonstrated that neo-nephrogenesis after resection was rapidly ceased by P4 in the kidney, although Six2+ cells were still present at that time point. One explanation for the difference in neo-nephrogenesis capabilities between P1x and P4x kidneys can be attributed to the localization of Six2+ cells in the kidney. In the P4 kidney, localization of Six2+ cells was restricted within RV where the nascent epithelial basement membrane becomes evident after the mesenchymal-epithelial transition of cap mesenchymal cells^30^,^42^. Basement membranes are specialized and organized extracellular matrices formed from various proteins, such as laminin, type IV collagen, and heparin sulphate proteoglycan, and are thought to play roles in cell adhesion, migration, and differentiation of tubular epithelial cells^42^,^43^. Particularly, during nephrogenesis, robust synthesis of basement membrane proteins occurs and the heterogeneity of its components contributes to the differentiation of tubular epithelial cells, eventually leading to the specification of distinct nephron segments^42^,^43^. Previous reports showed that the number of cells that underwent the epithelial-mesenchymal transition and subsequently transdifferentiated into myofibroblasts was extremely small in an injured adult kidney *in vivo*^44^,^45^, suggesting that epithelial cells cannot pass through the basement membrane. Other evidence that exogenous transplanted cells^17^ or endogenous bone marrow derived cells^18^,^19^ rarely repopulated into tubules directly during injury and repair suggests that the tubular basement membrane forms a barrier to prevent the cells from transmigrating into the tubules. Thus, it is possible that the basement membrane of RV prevented the Six2+ cells from migrating toward the resected site, and, consequently, these cells could no longer participate in neo-nephrogenesis in the P4x kidney. In contrast, because the cap mesenchyme was not surrounded by the basement membrane, Six2+ cells in the cap mesenchyme still present in the P1x kidney could migrate easily towards the resected region in response to various signals, not only for development, but also for injury and repair.

From an immunological point of view, our results demonstrated the infiltration of macrophages and monocytes into the re-growing regions of the P1x kidney to a lesser extent than observed in P4x kidneys. The reduction of macrophages and monocytes may support the proliferation of nephron and ureteric bud progenitor cells, leading to neo-nephrogenesis. The relationship between scar-free wound healing and regeneration in amphibians can be explained in the context of immunological competence^46^. Urodeles, such as newts and axolotls, and anurans, such as frogs and toads, differ widely both in these phenomena and in their immune systems. The former maintain their capacity to repair wounds and regenerate various organs throughout their life, while the latter lose both capacities with development. Salamanders possess a strong innate immunity, but their adaptive immunity is relatively deficient when compared to that of frogs and mammals^47^. On the other hand, frogs have a fully developed innate immunity and robust adaptive immunity in adulthood^48^. There are several lines of evidence to support the inverse relationship between immune competence and epimorphic regeneration, even in the fetal limb bud^49^ and the repair of skin wounds^50^ in mice, which develop adaptive immunity around birth^51^. Embryonic wounds exhibit scar-free healing with the reduction of infiltrated inflammatory cells, which contain fewer activated macrophages^52^. Neonatal skin wounds in PU.1-null mice, which lack macrophages and functioning neutrophils, are repaired and regenerated without scars or an inflammatory response, while wild-type mice exhibit significant scarring^53^. Neonatal myocardial infarction models demonstrate the opposite phenotype, in that macrophage-depleted mice form fibrotic scars, whereas wild-type mice regenerate the myocardium with minimum scarring^54^. In contrast, adult mice in which macrophages are pharmacologically depleted showed poor healing following myocardial infarction, with reduced angiogenesis and scar formation, resulting in high mortality due to left ventricular remodeling, subsequent to ventricular rupture^55^. In the context of transdifferentiation processes, adaptive immunity could exclude cells expressing antigens related to dedifferentiation. Macrophages are major players in innate immunity, and could be one of the main populations that orchestrate both regeneration with angiogenesis following amputation in various models^56^ and inflammation to recruit fibroblasts; the fibroblasts differentiate into myofibroblasts to deposit excessive collagen, and consequently form fibrotic scars. Macrophages in the wound could execute their mission in a context-dependent manner. Macrophages possess heterogeneous populations with distinct functional phenotypes, a subset of which perform the majority of macrophage function, but how they react following insult remains to be elucidated.

Although we demonstrated the possible contribution of Six2+ cells in CM on neo-nephrogenesis after resection, we realize our study is limited by the lack of direct evidence to support this contention. We cannot exclude the possibility that the cells originating from outside the kidney migrated and engrafted into the resected site, and eventually differentiated into nephron components. Lineage tracing of Six2+ cells using transgenic mice, in which tamoxifen dependent Cre recombinase is expressed under control of the *Six2* locus, may resolve this issue. Lineage tracing may also explain the precise process by which the new nephron is rebuilt after resection. However, nephrectomy of neonatal mice is technically challenging, and results in a high mortality rate after surgery. Modification of the surgical procedure for neonatal mice will be required to answer this issue.

In conclusion, we demonstrate that the rat kidney in early neonates retains the capability to accelerate neo-nephrogenesis after resection insult; however, it loses this potential by postnatal day 4. This loss of potential coincides with the disappearance of Six2+ cells. Six2+ cells are strong candidates for this phenomenon, and lineage analysis of this population may provide further insight into this issue. This partial nephrectomy in early neonates can provide a model to understand the molecular mechanisms regulating neo-nephrogenesis as well as for the prevention of scar formation and to uncover novel interventions for chronic kidney disease.

## Materials and Methods

### Animal experiments

All experiments were performed according to the animal experiment guidelines issued by the Animal Care and Use Committee at the Kyoto Prefectural University of Medicine (approval number M26-302). Partial nephrectomies were performed on neonatal rats (Wistar Rat) at postnatal days 1 and 4 (Supplementary Movie S1). Neonates were anaesthetized by cooling on an ice bed for 3–5 min as described previously^57^. An incision was made in the right flank, and the right kidney was gently exposed. Iridectomy scissors were used to resect approximately 10% in weight of the right kidney at the inferior pole. Following partial resection, neonates were transferred from the ice bed to room temperature; thereafter, the incisions were continuously sutured with 7-0 Proline. Neonates were then held in the operator’s hand for warmth for several minutes until recovery. The entire procedure lasted approximately 20 min. Of P1 neonates, 90% survived the surgical procedure: all deaths occurred either during or on the day of the surgery. Our surgical protocol involved weighing resected kidneys to mark the limit of resection, which ensured the reproducibility of the procedure. All-trans retinoic acid (Sigma-Aldrich, St. Louis, MO) was dissolved in 0.9% NaCl with 10% ethanol and 10% BSA. Reagents (10 mg/kg) were administered intraperitoneally once daily for 14 days after resection.

### Tissue preparation

Rats were anesthetized and sacrificed, and kidneys were removed at the indicated time points (0, 1, 2, 4, 7, 14, 28 days post-resection). For paraffin sections, kidneys were fixed with 4% (vol/vol) paraformaldehyde (Wako Pure Chemical Industries, Ltd., Osaka, Japan), paraffin-embedded, and cut into 4 μm sections. For frozen sections, kidneys were fixed with 4% paraformaldehyde for 2 h on ice, incubated in 30% (vol/vol) sucrose in PBS at 4 °C for overnight, embedded in optimum cutting temperature compound (Sakura FineTek Japan co., Ltd., Tokyo, Japan), and cut into 5 μm sections.

### Histology and immunohistochemistry

PAS and H&E staining were performed according to standard procedures. Paraffin sections were placed in citrate-buffered solution (pH 6.0) and boiled for 10 min for antigen retrieval. Endogenous peroxidase was blocked with 3% hydrogen peroxide. Samples were blocked with 3% BSA in PBS and incubated with primary antibodies, namely Rabbit anti–Podocin (1:500; ab93650, Abcam, Cambridge, MA), Aquaporin 1 (1:500; ab9566, Abcam), CD68 (1:500; ab31630, Abcam), PHH3 (1:500; #9706s, Cell Signaling Technology, Danvers, MA), and the lectin, *Dolichos biflorus* Agglutinin (DBA, 1:500; Vector Labs, Burlingame, CA). Diaminobenzidine substrate (Dako, Hamburg, Germany) was used for the color reaction. Sections were counterstained with hematoxylin. Secondary antibody alone was consistently negative on all sections.

### Immunofluorescence

Sections were rehydrated and permeabilized with 0.2% Triton X-100 in PBS for 5 min. Samples were blocked with 3% BSA in PBS and incubated with primary antibodies (anti-Six2, 1:500; 11562-1-AP Proteintech, Chicago, IL, anti-Collagen IV, 1:500; ab6566 Abcam, anti-Pax2; 1:500; ab79389, Abcam). Samples were then incubated with secondary antibodies, Alexa Fluor 594 conjugate and Zenon IgG labeling Kit Alexa Fluor 488 (Thermo Fisher Scientific, Waltham, MA), for 1 h. Nuclear counterstaining was performed using DAPI or DRAQ5 (BioStatus, Leicestershire, UK), followed by mounting in Prolong-Gold (Thermo Fisher Scientific). Images were obtained by confocal (FV1000; Olympus) or standard (IX-71, DP-73; Olympus) microscopy.

### Quantifying of histology

The glomerular number was counted and the amount of AQP1+ tubular epithelia was quantified by immunohistochemical staining for podocin and AQP1, respectively. Because, in rodents, kidney tissue grows until 42 days after birth and the generated tissue makes the resected site unclear, we needed to determine the area to be quantified. To overcome this limitation, we drew a fan-shaped area, which included the resection site, with an 80° angle and its vertex set at the top of the papilla (Supplementary Fig. S6). We counted the glomerular number and quantified the AQP1+ epithelia within this marked area by using Image J software (National Institutes of Health), and compared these to those of normal controls at each time point.

The quantification of the collecting duct was performed by immunostaining for DBA. Collecting ducts usually radiate towards the kidney surface from the renal papilla; thus, we drew a curved line at the cortico-medullary junction, and the DBA+ tubules that intersected at that line were counted (Supplementary Fig. S7). PHH3+ proliferating cells or TUNEL+ apoptotic cells were counted from five of 25 consecutive non-overlapping fields in each kidney under high magnification with a 200x objective. These five fields were randomly selected in a blind fashion.

### RNA isolation and real-time quantitative PCR

To isolate and amplify transcripts in the generating kidney after resection, we roughly dissected a fourth of the kidney, which contained the generation plane. Total RNA was extracted from resected and age-matched control kidneys at 0, 1, 2, 4, and 7 days post-surgery by using TRIzol (Life Technologies, Carlsbad, CA, USA) and Direct-zol™ RNA MiniPrep (Zymo Research), followed by cDNA synthesis using Prime Script kit (Takara Bio Inc., Shiga, Japan). Real-time detection of the PCR products was performed using the SYBR Green Master Mix (Kapa Biosystems), and all reactions were conducted in duplicate. Primers for targets and reaction protocols are listed in Supplementary Table 1.

### Statistical analysis

Results are expressed as mean ± standard error (SE). Each experiment was performed with n ≥ 3 per group. Quantification was performed using ≥10 high-power field images for each kidney. Statistical analysis was performed using the unpaired t-test. *P* values of <0.05 were considered significant.

## Additional Information

**How to cite this article**: Kirita, Y. *et al.* Preserved Nephrogenesis Following Partial Nephrectomy in Early Neonates. *Sci. Rep.* **6**, 26792; doi: 10.1038/srep26792 (2016).

## Supplementary Material

Supplementary Figures

Supplementary Video
