# Supplementary material for: Preserved Nephrogenesis Following Partial Nephrectomy in Early Neonates
Source: Sci Rep. 2016 May 31;6:26792. doi: 10.1038/srep26792 (PMC4886582; doi:10.1038/srep26792)
Supplement: Supplementary Figures [file srep26792-s1.pdf]

Supplementary Information

Preserved Nephrogenesis Following Partial Nephrectomy in Early Neonates

Yuhei Kirita[1], Daisuke Kami[2], Ryo Ishida[1], Takaomi Adachi[1], Keiichi Tamagaki[1],

Satoaki Matoba[3], Tetsuro Kusaba[1], Satoshi Gojo[2]*

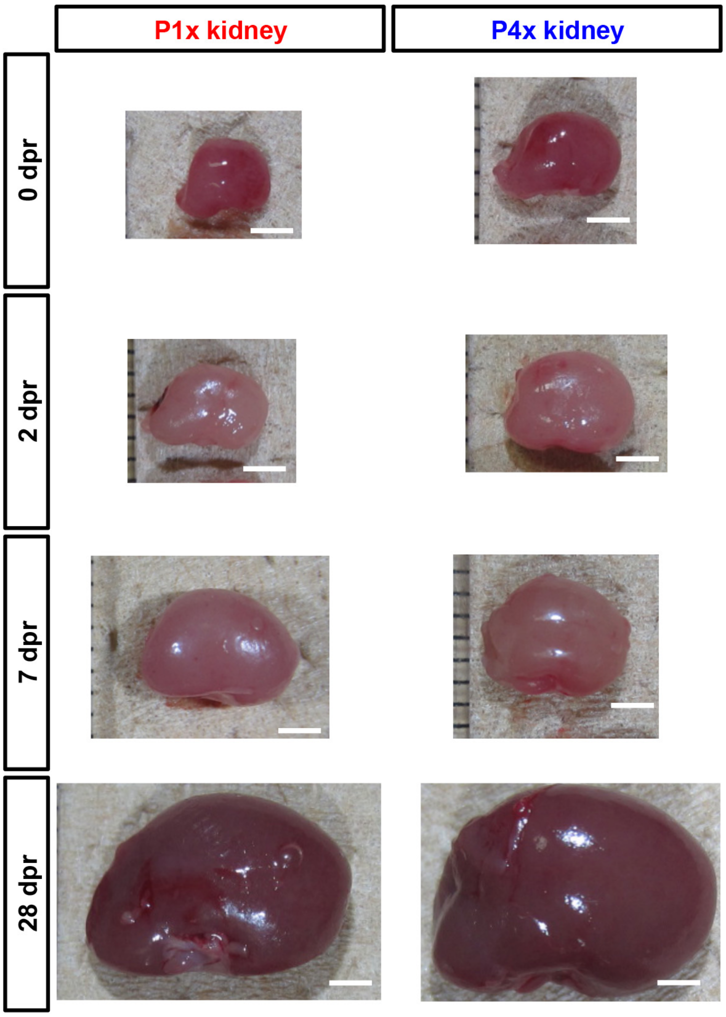

Supplementary Figure S1

Macroscopic pictures of resected kidneys. The P1x kidney shows the bulge of the newly formed tissues from the edge of the wound (scale bars, 2 mm).

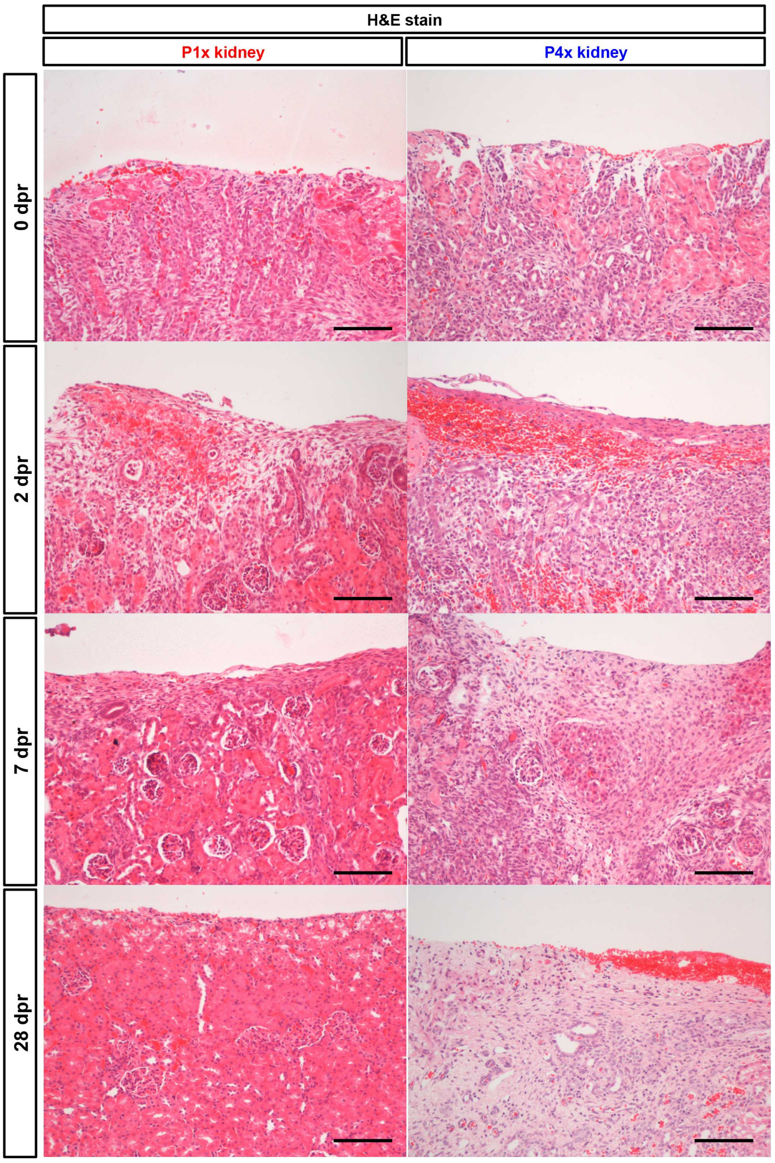

Supplementary Figure S2

H&E staining of resected kidneys around the resection plane. HE staining demonstrates that the surface of resected site is covered with a blood clot and infiltrated by inflammatory cells in both P1x and P4x kidneys at 2 dpr. Infiltration by inflammatory cells reduces rapidly in P1x kidney; in contrast, this reduction is slow and scar formation was prominent in the P4x kidney. (scale bars, 100 μm).

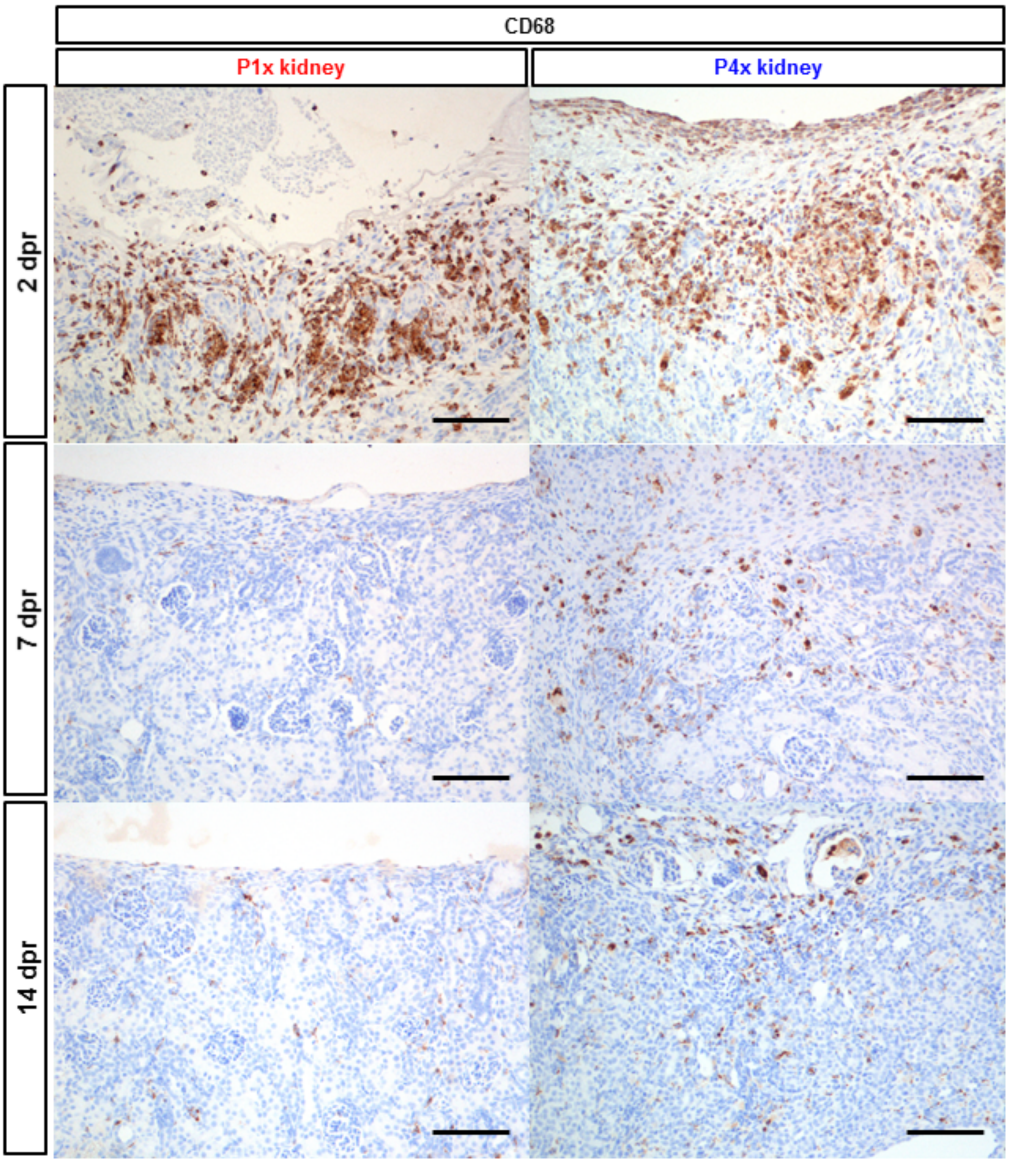

Supplementary Figure S3

Macrophage infiltration into resected kidneys. Immunohistochemistry for CD68 as a macrophage marker at 2, 7, and 14 dpr. Substantial CD68+ macrophage infiltration was observed at 2 dpr in both the P1x and P4x kidneys. At 7 dpr, CD68+ macrophages did not exist in the P1x kidney; however, some cells were still remain in the P4x kidney. Scale bars, 100 μm.

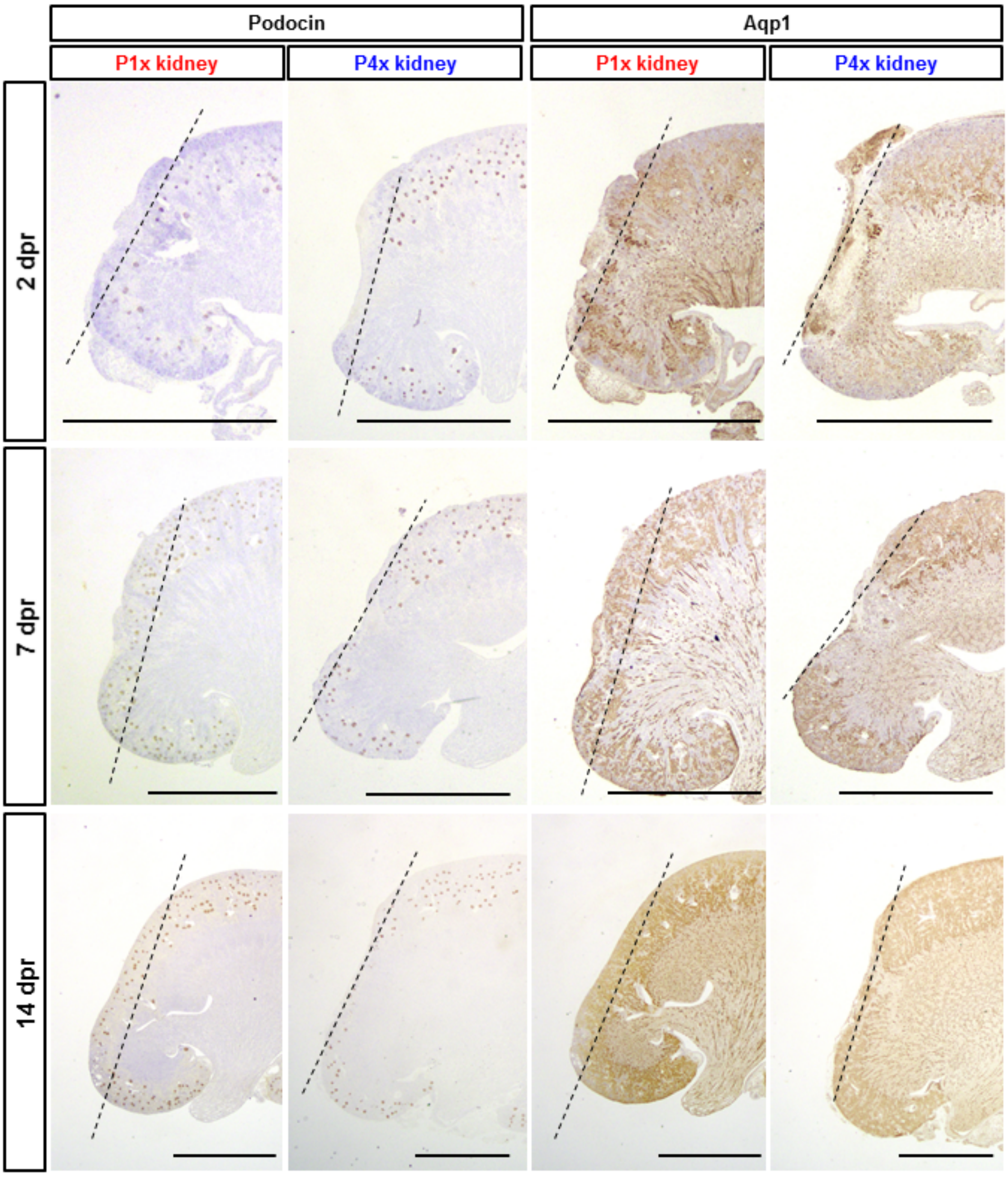

Supplementary Figure S4

Immunohistochemistry of Podocin and Aqp1 at 2, 7, 14 dpr. In the P1x kidney, the parenchymal tissue gradually covers the resected surface and contains glomeruli and tubules. In contrast, the resected surface of the P4x kidney is not covered by the parenchymal tissue. Scale bars, 2 mm.

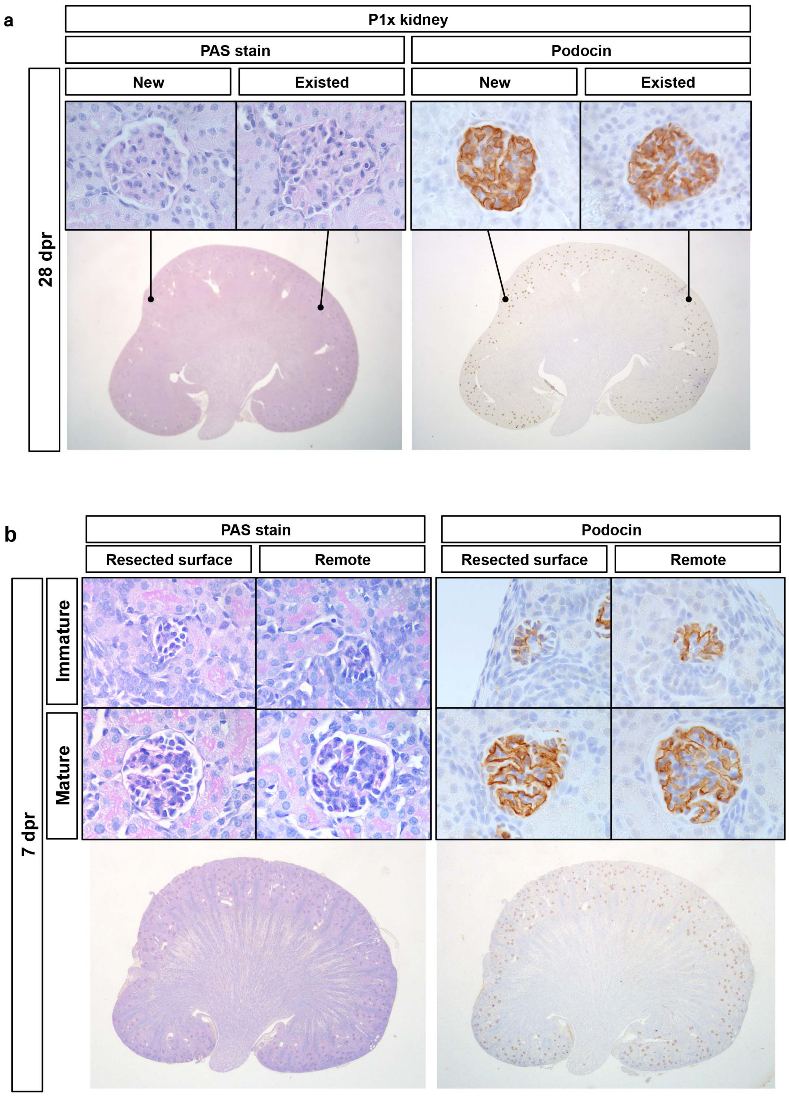

Supplementary Figure S5

PAS staining and immunohistochemistry of Podocin at 28 and 7dpr of P1x kidney. (a) There was no differences in histological finding and podocin expression between newly generated glomeruli after resection and that of remote region of P1x kidney at 28dpr. (b) Several developing glomeruli defined as less podocin staining existed in both resected area and remote region of P1x kidney at 7dpr. There was no histological differences between those.

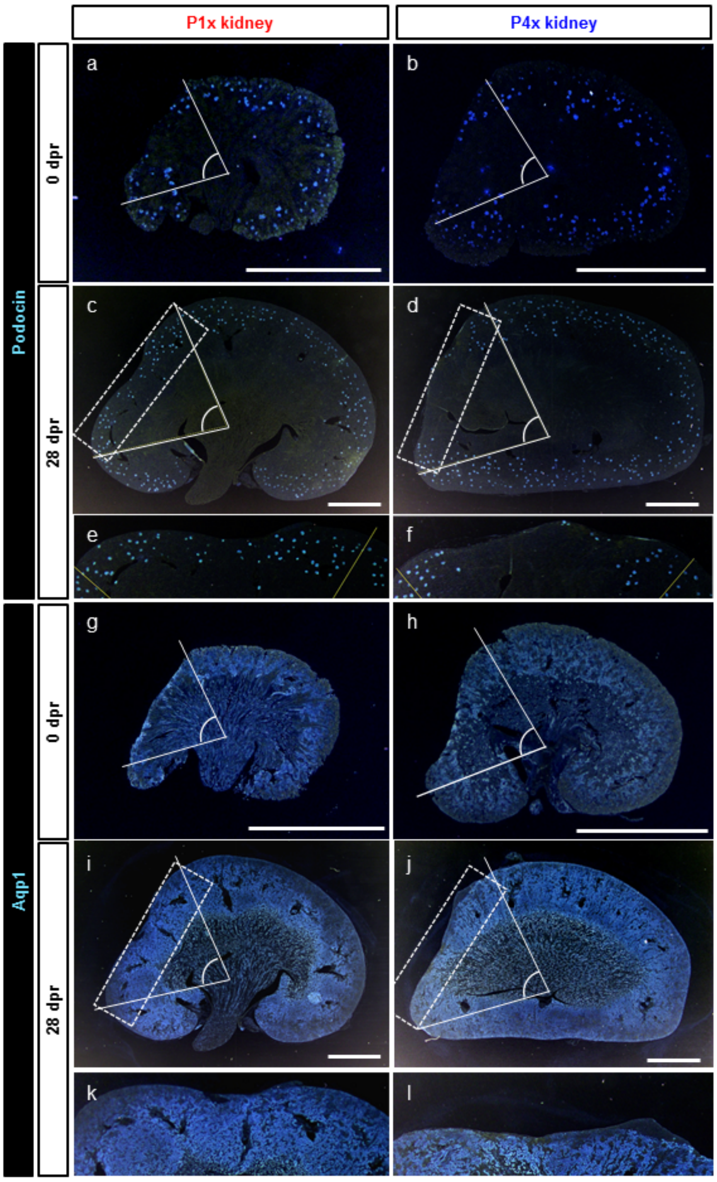

Supplementary Figure S6

Determination of area for quantification of glomerulus number and cortical area. Immunohistochemistry for Podocin and Aqp1. The arcs indicate 80 degrees—the angle for quantifying glomeruli and tubules over the wound surface. (a, b, g, h) Immediately after nephrectomy in both P1x and P4x kidneys, the angles made by rays to the lateral

and medial edges of the wound line, with the centre of the renal pelvis as the vertex, in the coronal slice with maximal cross-sectional area, are around 80 degrees, with no significant difference between the two groups. (c, d, e, f) At 28 dpr in the quantitative area (e, f; dotted squares in c, d), there are significant number of glomeruli in the P1x kidney, but few in the P4x kidney. (i, j, k, l) At 28 dpr in the quantitative area (k, l; dotted squares in i, j), the cortical area in the P1x kidney is thicker than that in the P4x kidney. Scale bars, 2 mm

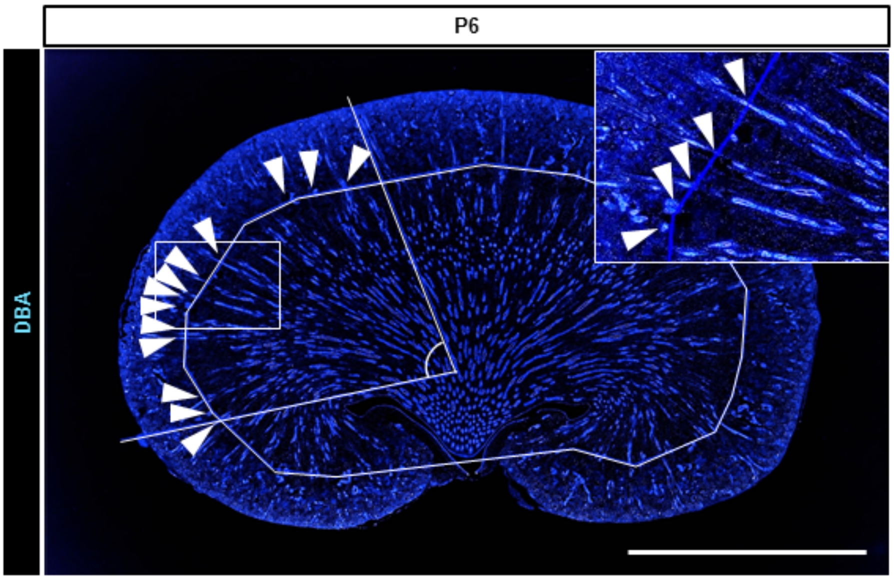

Supplementary Figure S7

Determination of area for quantification of DBA+ tubules. White-lined circles indicate the cortico-medullary border. Arrowheads indicate DBA+ tubules, which intersect at the cortico-medullary border. Scale bar, 2 mm. The arcs indicate 80°

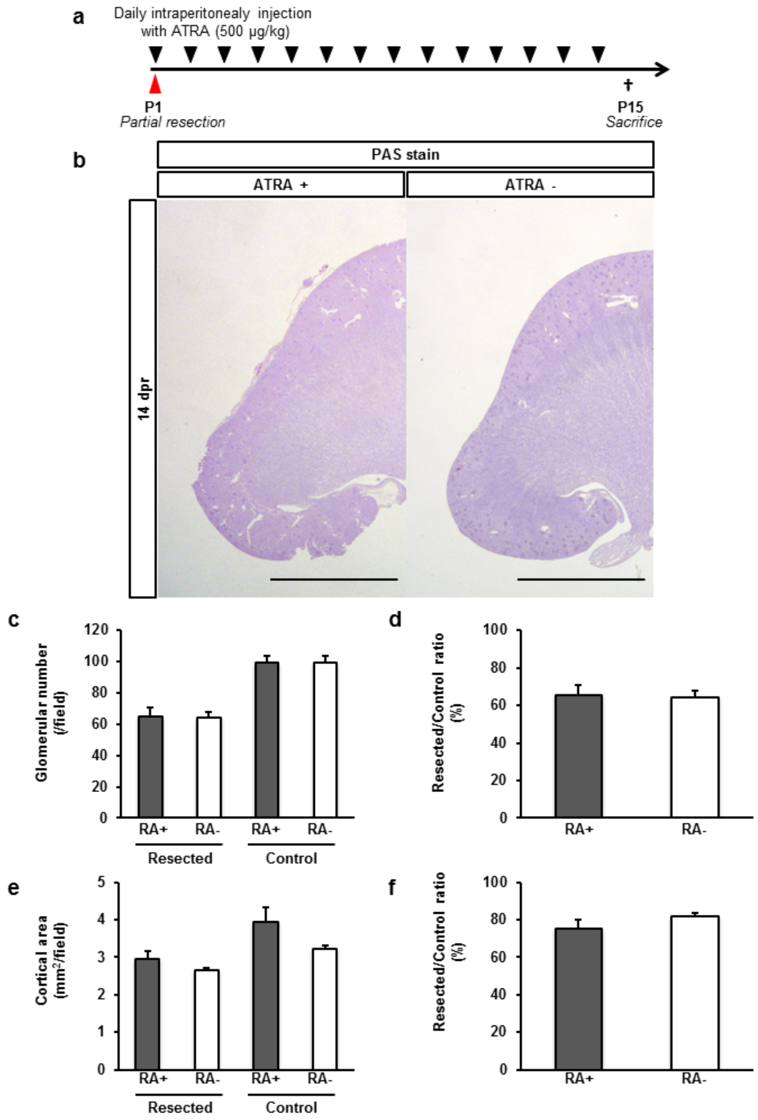

Supplementary Figure S8

Daily retinoic acid administration did not enhance neonephrogenesis in the renal cortex, including in the glomerulus. (a) Experimental scheme. All-trans retinoic acid (ATRA) was intraperitoneally administered daily for 14 days after partial nephrectomy, and pups were sacrificed at 14 days post resection. (b) PAS staining showed no significant histological change caused by daily ATRA injection. (c) Glomerular number in the

resection plane in the maximum cross sectional surface. (d) Resected/control ratio of the glomerular number. (d) Cortical area in the resection plane at the maximal cross sectional surface. (f) Resected/control ratio of the cortical area. Data shown indicate mean of sample averages ± SE (n = 3)

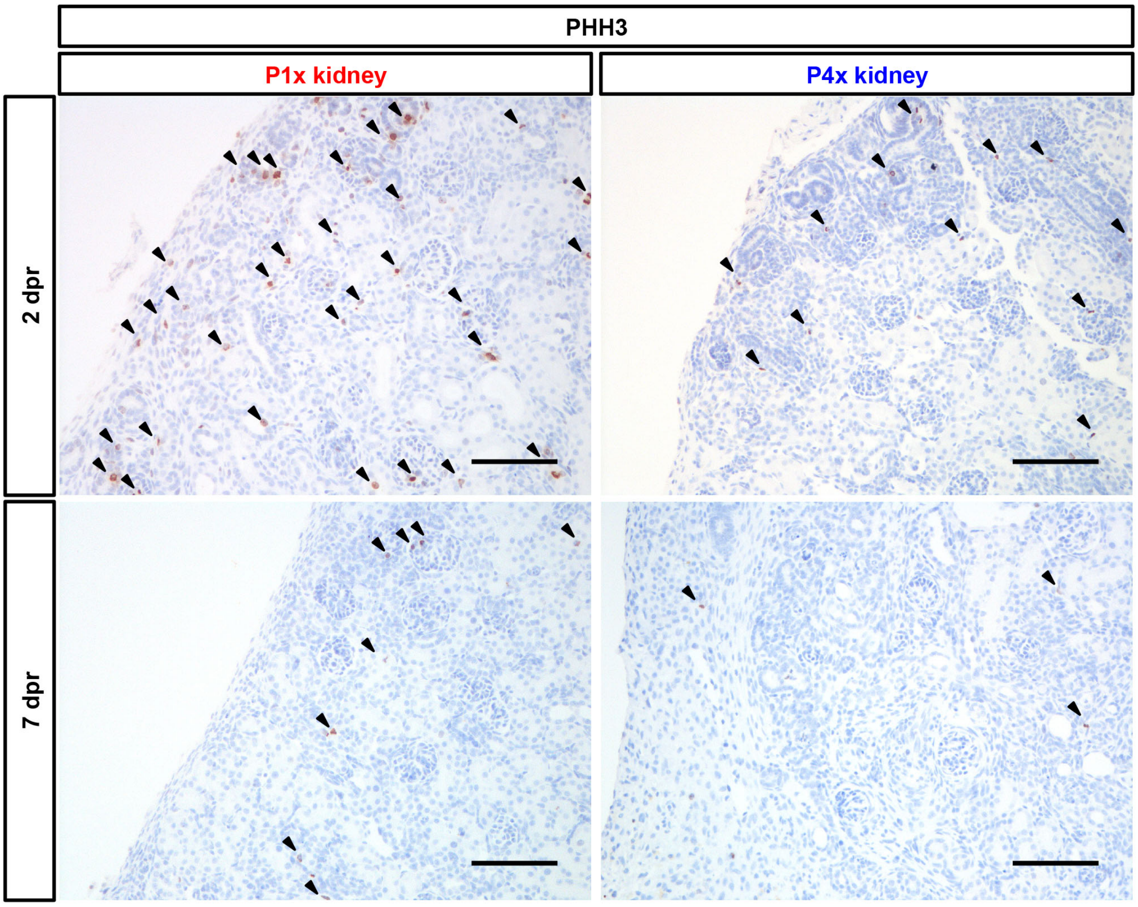

Supplementary Figure S9

The P1x kidney preserves proliferative capacity after partial nephrectomy. PHH3+ cells were prominent in P1x kidney at both 2dpr and 7dpr compared with those of P4x kidney. Arrowheads indicate PHH3+ cells. Scale bars, 100 µm

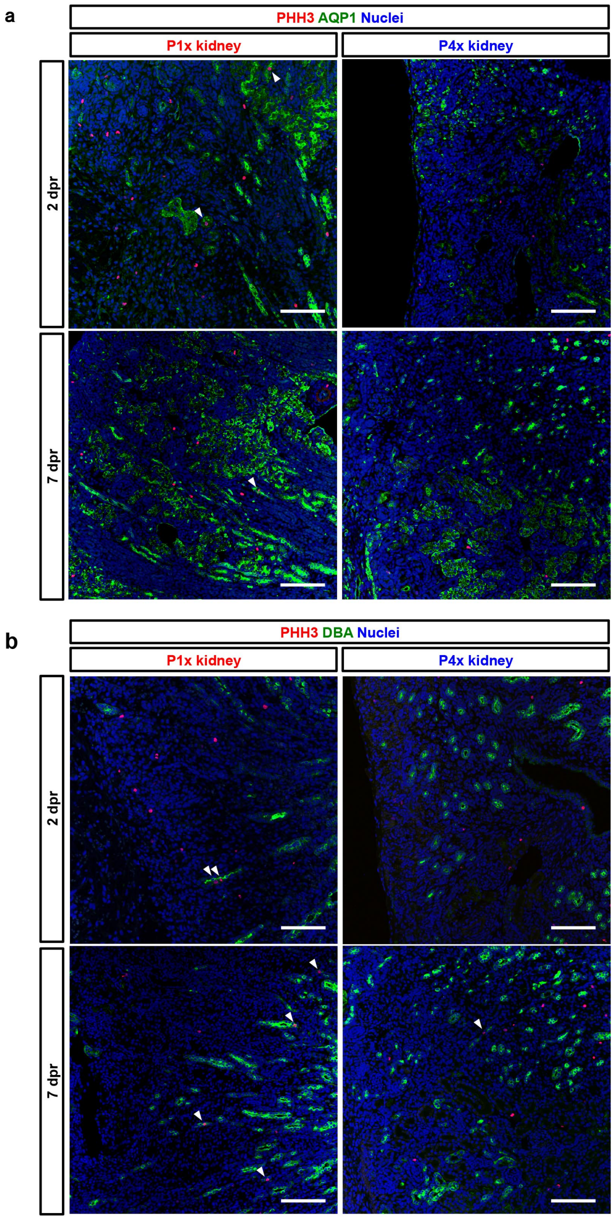

Supplementary Figure S10

Analysis of proliferating proximal tubular epitherial cells and collecting duct cells after resection. (a) Co-staining of PHH3 and AQP1 for detection of proliferating proximal tubular epithelia (arrowheads). (b) Co-staining of PHH3 and DBA for detection of proliferating collecting duct cells (arrowheads).Scale bars, 100 μm.

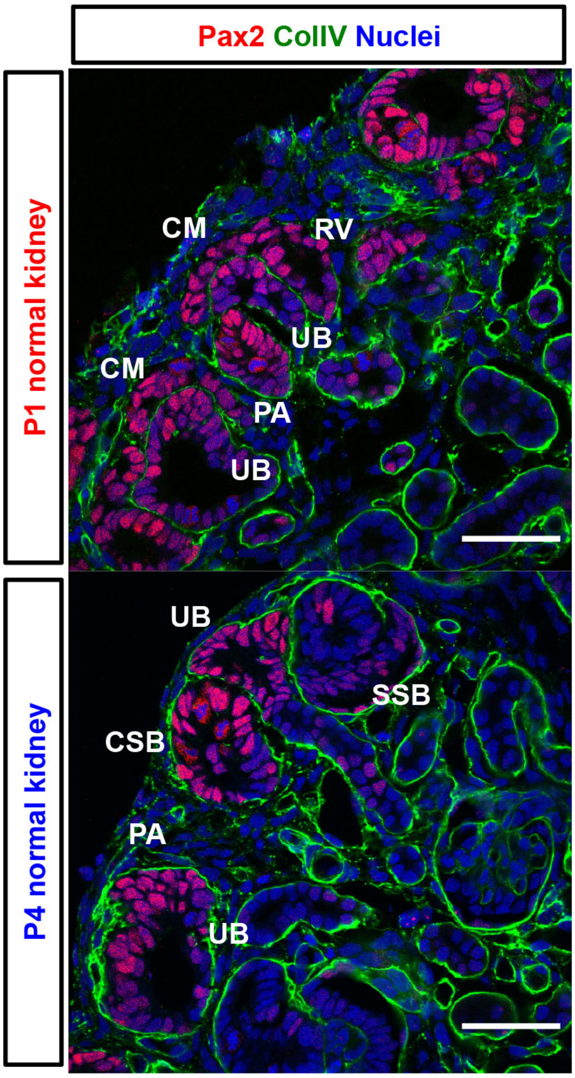

Supplementary Figure S11

Immunofluorescence staining for Pax2 and ColIV in P1 and P4 kidneys. Scale bars, 50μm. Pax2+ cells are present in the peripheral layer in both P1 and P4 kidneys; however, its expression in the P4 kidney is weaker than that in the P1 kidney. CM, cap mesenchyme; PA, pretubular aggregate; RV, renal vesicle; CSB, Comma-shaped body; SSB, S-shaped body; UB, ureteric bud.

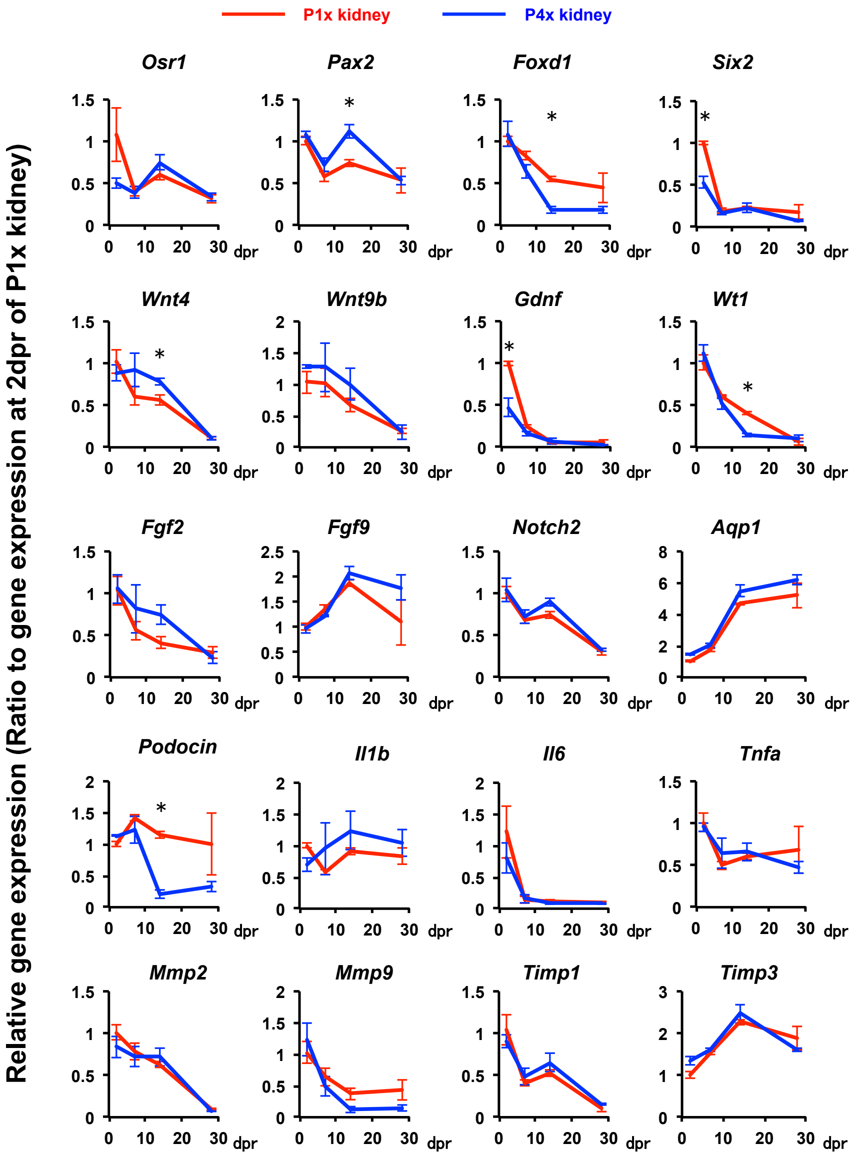

Supplementary Figure S12

Gene expression profiles of markers for kidney development (*Osr1, Pax2, Foxd1, Six2, Wnt4, Wnt9b, Gdnf, Wt1, Fgf2, Fgf9, Notch2, Aqp1 and Podocin*), inflammation (*Il1b, Il6 and Tnfa*) and tissue fibrosis (*Mmp2, Mmp9, Timp1 and Timp3*) in P1x and P4x with time following the resection. Data represent mean ± SE (n = 3). *Statistically significance between P1x and P4x kidney at each time point defined as P<0.05.

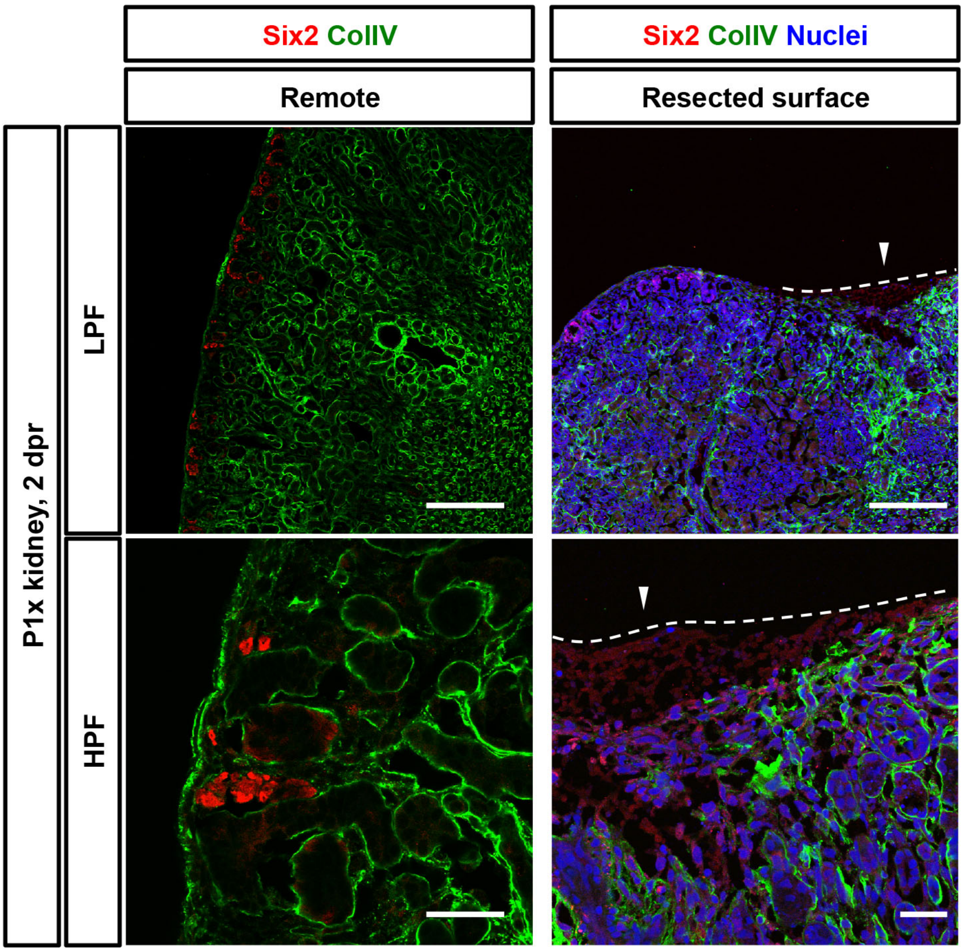

Supplementary Figure S13

Immunofluorescence staining for Six2 and ColIV at 2dpr of P1x kidney. (a) Six2 expression was preserved in the area surrounding the wound compared with remote region at 2dpr of P1x kidney. Arrowheads indicates the resected area. Dashed line indicate the clot area at 2dpr of P1x kidney. Scale bars in low power field (LPF), 200 µm; in high power field (HPF), 50µm. (b)

Gene name	Primer Sequence	Size(bp)
rGAPDH	CCTGCACCACCAACTGCTTAGC<br>GCCAGTGAGCTTCCCGTTCAGC	239
rSix2	CTCACCACCACGCAGGTCAG<br>CGTCCTCGGAACTGCCTAGC	169
rNotch2	GAGTGTCTGAAGGGCTACGC<br>CTGCCCATTGTTTACACACG	198
rAQP1	ATTGCAGCGTCATGTCTGAG<br>GAACTAGGGGCATCCAAAC	99
rPodocin	GCACAAAGACAGGCCAAA<br>ACTCAGAGGCAGCTTTTTCCC	61
rWNT4	CTGGAGAAGTGTGGCTGTGA<br>GGACTGTGAGAAGGCTACGC	108
rFoxd1	CTACTCGTACATCGCGCTCA<br>CTCCCGGTAGTAAGGGAAGC	112
rPax2	TACACTGATCCTGCCCACATTAGA<br>GGATAGGAAGGACGCTCAAAGACT	196
rWNT9b	TGAAGCAGTGTGACCTCCTG<br>GCGTACAGGAAGGCTGTCTC	208
rOsr1	TCTTTCCTTCAGGCAGTGAATG<br>TCTCTGGCTTAGGGTGAATGAC	241
rHoxa11	GATCTGCACCCAAACCTGAG<br>CAACCAGCAAAACCTCTGGA	161
rFgf2	GAACCGGTACCTGGCTATGA<br>CCGTTTTGGATCCGAGTTTA	182
rFgf9	CGCAGTCACGGACTTGGATC<br>ACACCACGAATGCTGACCAG	183
rIl1b	TCACTCATTGTGGCTGTGGAGAAG<br>CACACACTAGCAGGTCGTCATCAT	154
rIl6	CCGGAGAGGAGACTTCACAG<br>ACAGTGCATCATCGCTGTTC	161
rTnfa	TGATCCGAGATGTGGAACTG<br>CGAGCAGGAATGAGAAGAGG	105
rMmp2	ACACTGGGACCTGTCACTCC<br>ACACGGCATCAATCTTTTCC	193
rMmp9	ATATGGTTTCTGCCCCAGTG<br>AGTTGCCCCCAGTTACAGTG	224
rTimp1	TCCCCAGAAATCATCGAGAC<br>TCAGATTATGCCAGGGAACC	250
rTimp3	TGTGCAACTTTGTGGAGAGG<br>GTACCCGAAATTGGAGAGCA	173
rWt1	TGCCACACCCCTACCGACAGTT<br>CTTCAAGGTAGCTCCGAGGTTCATC	141

Supplementary Table S1

Primers used in quantitative real-time PCR

Supplementary Movie S1

Procedure of partial nephrectomy in neonatal rat.
